# Causal association between serum total bilirubin and cholelithiasis: a bidirectional two-sample Mendelian randomization study

**DOI:** 10.3389/fendo.2023.1178486

**Published:** 2023-07-04

**Authors:** Yang Sun, Shaojie Yang, Wanlin Dai, Zhuyuan Zheng, Xiaolin Zhang, Yuting Zheng, Jingnan Wang, Shiyuan Bi, Yunlong Duan, Shuodong Wu, Jing Kong

**Affiliations:** ^1^ Biliary Surgery (2nd General) Unit, Department of General Surgery, Shengjing Hospital of China Medical University, Shenyang, Liaoning, China; ^2^ Innovation Institute of China Medical University, Shenyang, China

**Keywords:** Mendelian randomization, total bilirubin, cholelithiasis, causal association, bidirectional, two-sample

## Abstract

**Background:**

Observational studies about the association between serum total bilirubin and cholelithiasis are inconsistent. Hence, it is essential to reevaluate the association between serum total bilirubin and cholelithiasis and to verify whether such association is causal or not.

**Methods:**

We selected single-nucleotide polymorphisms (SNPs) that are strongly associated with exposure as instrumental variable and conducted a bidirectional two-sample Mendelian randomization (MR) study to explore the causal association between serum total bilirubin and cholelithiasis. We implemented the inverse-variance weighted approach as a primary analysis to combine the Wald ratio estimates. Four additional analyses, namely, MR-Egger regression, weighted median, weighted mode, and MR–pleiotropy residual sum and outlier (PRESSO), were utilized to investigate the causal association and the influence of potential pleiotropy.

**Results:**

A total of 116 SNPs were selected as valid instrumental variables to estimate the causal association of serum total bilirubin on cholelithiasis, and causal association between genetically determined serum total bilirubin and cholelithiasis was demonstrated [beta = 0.10; 95% confident interval (CI), 0.07 to 0.14; p < 0.001]. Likewise, the other methods, namely, the weighted median (beta = 0.12; 95% CI, 0.08 to 0.15; p < 0.001), MR-Egger (beta = 0.11; 95% CI, 0.08 to 0.15; p < 0.001), weighted mode (beta = 0.11; 95% CI, 0.08 to 0.15; p < 0.001), and MR-PRESSO approaches, further confirmed that this result (p = 0.054) indicates similar results. In addition, seven SNPs were selected as instrumental variable to estimate causal association of cholelithiasis on serum total bilirubin, and the result supported the causal effect of cholelithiasis to serum total bilirubin (beta = 0.12; 95% CI, 0.09 to 0.15; p < 0.001). At the same time, the other methods, namely, the weighted median (beta = 0.10; 95% CI, 0.06 to 0.13; p < 0.001), MR-Egger (beta = 0.12; 95% CI, 0.07 to 0.18; p = 0.007), weighted mode (beta = 0.09; 95% CI, 0.03 to 0.14, p = 0.019), and MR-PRESSO methods, further confirmed this result (p < 0.001).

**Conclusion:**

Our MR study revealed that the serum total bilirubin was causally associated with the risk of cholelithiasis, and the genetic predisposition to cholelithiasis was causally associated with the increased serum total bilirubin levels.

## 1 Background

The high number of individuals affected by cholelithiasis (gallstone disease), estimated to be 10%–20% of the adult population, has posed a major financial strain on society around the world (1). An enormous amount of work has gone into determining the risk factors that lead to the development of cholelithiasis to alleviate this health burden. Most gallstones are found in the gallbladder (cholecystolithiasis). Occasionally, however, they pass through the cystic duct into the common bile duct and/or intrahepatic bile ducts, resulting in choledocholithiasis and/or hepatolithiasis (2).

It is well known that bilirubin is a degradation product of heme after aging red blood cells break down. Calcium and biliary bilirubin can combine to produce calcium bilirubinate salts, which can then crystallize and cause pigment gallstones (3). In addition, cholesterol gallstones, the most prevalent type of gallstones in the Western world, commonly have a core of calcium bilirubinate (4). Cholesterol gallstones may contain up to 30% calcium bilirubin, whereas bile pigment gallstones consist primarily of bilirubin (3, 5).

Several observational studies have demonstrated an association between elevated serum total bilirubin and an increased risk of gallstone disease (6–8). However, the research conducted by Shrestha et al. found no association between higher serum total bilirubin and the development of gallstone in subjects with β-thalassemia (9). These observational results are inconsistent, so it is essential to reevaluate the association between serum total bilirubin and cholelithiasis and verify whether such association is causal or not. Evaluating the association between serum total bilirubin and cholelithiasis can influence disease prevention and treatment strategies.

To estimate the causal relationship between an exposure and an outcome, Mendelian randomization (MR) makes use of the random allocation of genetic variants during conception as instrumental variables (IVs) (10). It is a genetic approach that utilizes a non-experimental design to determine whether or not an exposure caused a given outcome. Observational studies are prone to bias from unmeasured confounding factors and reverse causation, but MR study avoids these issues (11). Consequently, MR has been widely used to establish causal association between potential risk factors and diseases.

Notably, Stender et al. have tried to make use of one ample MR study and prospective study to estimate the association of bilirubin with symptomatic gallstone disease. They came out of the conclusion that both observationally and genetically elevated levels of plasma bilirubin are associated with an increased risk of symptomatic gallstone disease and that extreme bilirubin level is a causal risk factor for symptomatic gallstone disease (12). However, the MR study part was conducted with one sample and just one SNP, which led to the insufficiency of their study.

In our study, we utilized a bidirectional and two-sample MR study to reevaluate the possible bidirectional causal relationship between serum total bilirubin and the risk of gallstone disease.

## 2 Materials and methods

### 2.1 Data sources, study design, and instrumental variable identification

This bidirectional two-sample MR study focused on the causal relationship between serum total bilirubin and cholelithiasis and utilized summary statistics from two Genome-wide association studies (GWASs).

The summary GWAS statistics of cholelithiasis that we used was from FinnGen (https://www.finngen.fi/en). This GWAS consisted of 214,167 participants of European descent (19,023 cases and 195,144 controls), and 16,380,452 SNPs were included. Cholelithiasis in FinnGen GWAS is defined as International Classification of Diseases (ICD) codes for cholelithiasis (ICD-8:574, ICD-9:574, and ICD-10:K80), which consists of cholecystolithiasis, cholelithiasis, colic (recurrent) of gallbladder, gallstone (impacted) of cystic duct or/and gallbladder, choledocholithiasis, gallstone (impacted) of bile duct NOS, common duct and/or hepatic duct, hepatic cholelithiasis and/or colic (recurrent), and other cholelithiasis.

The GWAS summary statistics of serum total bilirubin was from UK Biobank (http://www.nealelab.is/uk-biobank). This GWAS consisted of 342,829 participants of European descent, and 13,585,986 SNPs were included.

**Figure 1:**
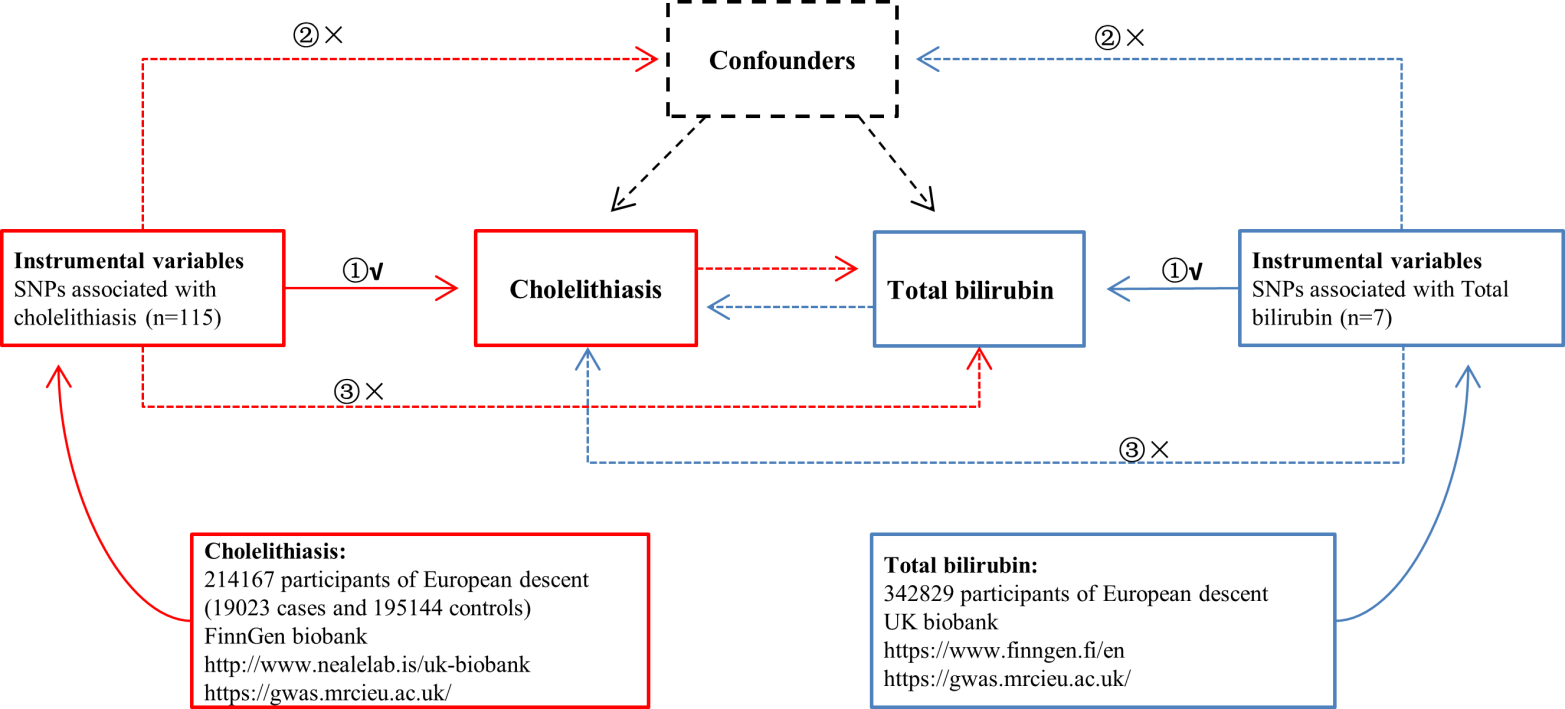
Overview of the study design in this bidirectional MR study. **①** The IVs are associated with the chosen exposure; **②** the IVs are not associated with the chosen outcome *via* a confounding pathway; **③** IVs are associated with the chosen outcome exclusively through exposure.

**Figure 1** provides the summary information of the relevant GWASs.

The IVs used in MR analyses must satisfy the following three fundamental assumptions (**Figure 1**).

(1) The IVs are associated with the chosen exposure.

(2) The IVs are not associated with the chosen outcome *via* a confounding pathway.

(3) IVs are associated with the chosen outcome exclusively through exposure.

**Figure 2:**
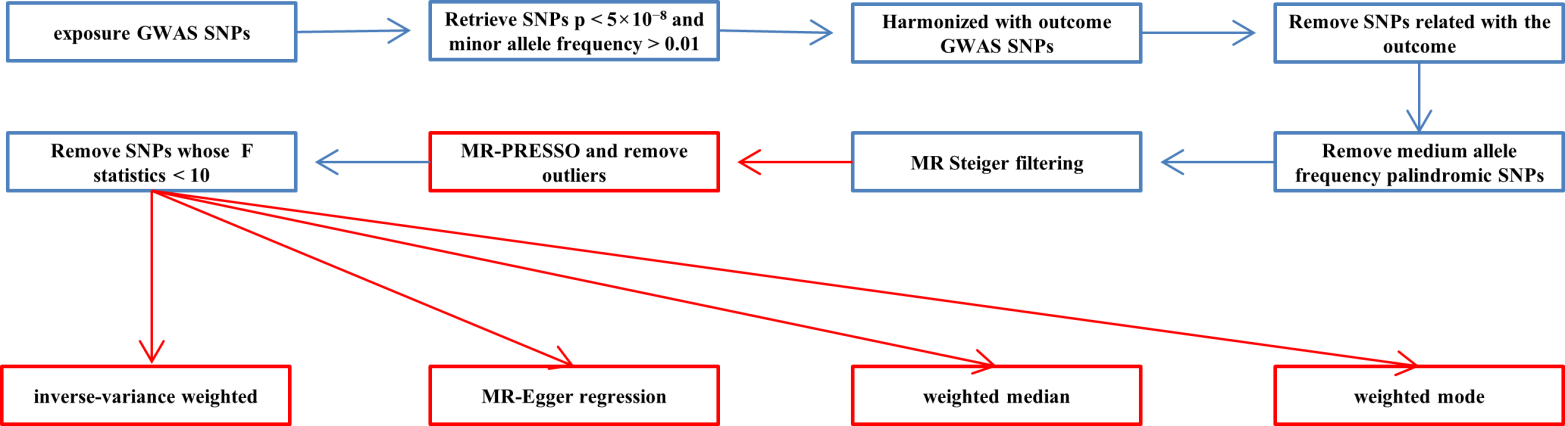
The process of selecting effective SNPs and statistical analysis.

The following procedures were used to select single-nucleotide polymorphisms (SNPs) from GWASs as IVs (**Figure 2**).

(1) SNPs reaching from corresponding GWAS whose p-value < 5×10^−8^ and minor allele frequency > 0.01 were included;

(2) Exposure SNPs harmonized with outcome SNPs, and SNPs that are related with the outcome were removed.

(3) To prevent duplicate counting and skewed causal effect estimations, we used a clumping procedure implemented in R software to detect independent variants, with a linkage-disequilibrium threshold of r^2^ < 0.001 within a 10,000-kb window in the European 1,000 Genomes Project Phase 3 reference pane (13).

(4) Palindromic SNPs that have a medium allele frequency (allele frequencies between 0.42 and 0.58) were filtered out of the pool of IVs, and a palindromic SNP is an SNP with the A/T or G/C allele.

(5) To eliminate SNPs with the invalid causal direction, MR Steiger filtering was utilized.

(6) The MR–pleiotropy residual sum and outlier (MR-PRESSO) method was utilized to identify outliers that could potentially influence the results and to determine whether causal estimates alter after removing outliers. Outliers will be eliminated from the harmonized data.

(7) We next assessed the power of the remaining SNPs using the F-statistic (F = beta^2^/se^2^) (14) for each SNP and computed an general F-statistic for all SNPs. SNPs having less statistical significance (F-statistics < 10) would be eliminated.

### 2.2 Statistical analysis

To evaluate the causal effect of exposure on site-specific outcome mediated by IVs, Wald ratios were utilized. With the goal of establishing a causal connection between serum total bilirubin and cholelithiasis, we implemented the inverse-variance weighted (IVW) approach as a primary analysis to combine the Wald ratio estimates. It is the most efficient analysis method with valid IVs. The assumption of this method is that none of the chosen IVs exhibit horizontal pleiotropy. If a causal effect is detected using this approach, then researchers should conduct sensitivity analyses to estimate the robustness of their finding (10).

Four commonly used sensitivity analyses, namely, MR-Egger regression, weighted median, weighted mode, and MR-PRESSO, were utilized to investigate the causal association and the potential pleiotropy, which are the most commonly used robust methods (15).

MR-Egger regression permits all IVs to have pleiotropic effects; however, the pleiotropic effects should be independent of the IV–exposure associations (referred to as the instrument strength independent of direct effect assumption) (11).

For weighted median and weighted mode, the former method assumes that at least 50% of the variants are valid IVs (majority valid assumption), whereas the latter method assumes that a greater proportion of variants estimate the true causal effect than estimate any other quantity (plurality valid assumption). They make weaker assumptions about the invalid IVs and are more robust to outliers (16, 17).

MR-PRESSO was used to detect and remove the outliers. It is a variant of the IVW approach that first eliminates from the analysis those IVs whose variant-specific causal estimates differ substantially from those of other variables (18).

R^2^ that represents the proportions of variance explained by exposures were calculated using the following formula: R^2^ = 2β^2^MAF (1 − MAF) (19).

All cited statistical analyses were performed using R software (version 4.2.1) and corresponding R packages (TwoSampleMR and MR-PRESSO).

## 3 Result

### 3.1 Serum total bilirubin to cholelithiasis

The causal association between serum total bilirubin and cholelithiasis was investigated using a two-sample MR analysis. The identified IVs, which comprised 116 SNPs (as shown in **Table S1**), could account for 29.00% of the variance in serum total bilirubin levels.

Moreover, the minimum F-statistic of these IVs was 27.31, suggesting that all IVs were sufficiently effective for the MR analysis (F-statistic > 10).

**Figure 3:**
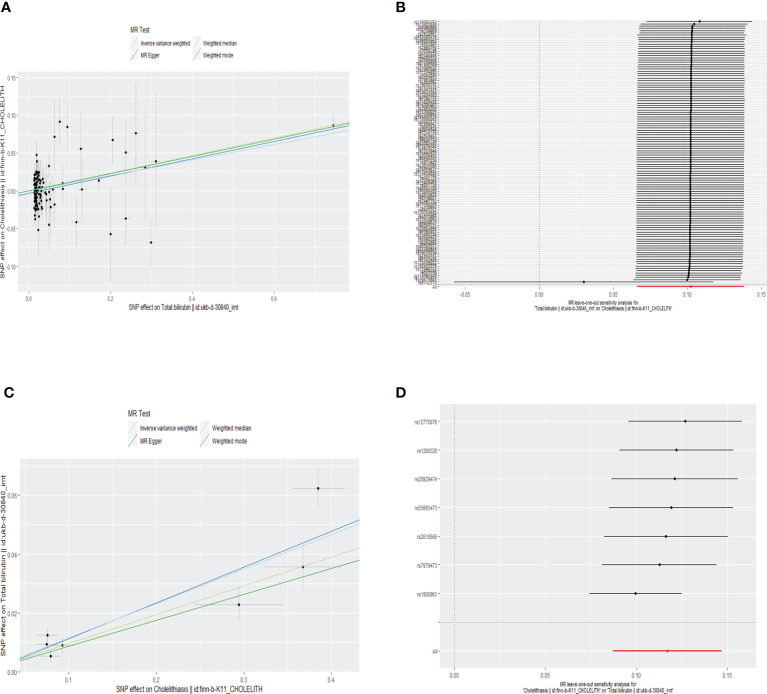
Scatter plot **(A)** and forest plot of leave-one-out result **(B)** of the relationship between genetically predicted total bilirubin on cholelithiasis. Scatter plot **(C)** and forest plot of leave-one-out result **(D)** of the relationship between genetically predicted cholelithiasis on total bilirubin.

According to random-effect IVW estimates, no causal association between genetically determined serum total bilirubin and cholelithiasis was demonstrated [beta = 0.10; 95% confident interval (CI), 0.07 to 0.14; p < 0.001]. Likewise, the other methods, namely, the weighted median (beta = 0.12; 95% CI, 0.08 to 0.15; p < 0.001), MR-Egger (beta = 0.11; 95% CI, 0.08 to 0.15; p < 0.001), weighted mode (beta = 0.11; 95% CI, 0.08 to 0.15; p < 0.001), and MR-PRESSO approaches, further confirmed that this result (p = 0.054) indicates similar results (as shown in **Table 1**). The scatter plot can be seen in **Figure 3A**.

**Table 1:** Effects of genetically predicted total bilirubin on cholelithiasis and cholelithiasis on total bilirubin in the MR analysis.

Exposure	Outcome	No. of SNP	Method	β (95% CI)	p-val	Egger_intercept	P-Egger_intercept
Total bilirubin	Cholelithiasis	116	Random-effect IVW	0.10 (0.07 to 0.14)	<0.001	−0.0031	0.098
			Weighted median	0.12 (0.08 to 0.15)	<0.001		
			MR-Egger	0.11 (0.08 to 0.15)	<0.001		
			Weighted mode	0.11 (0.08 to 0.15)	<0.001		
Cholelithiasis	Total bilirubin	7	Random-effect IVW	0.12 (0.09 to 0.15)	<0.001	−0.0007	0.852
			Weighted median	0.10 (0.06 to 0.13)	<0.001		
			MR-Egger	0.12 (0.07 to 0.18)	0.007		
			Weighted mode	0.09 (0.03 to 0.14)	0.019		

The MR-Egger regression result showed an intercept of −0.0031 with a p-value of 0.098, indicating no significant horizontal pleiotropy in the selected SNPs. Although the Cochran Q’s test shows heterogeneity in our data (Q = 171.829, P < 0.05), our results remain robust because the multiplicative random-effect IVW was used. The forest plot of leave-one-out result can be seen in the **Figure 3B**.

### 3.2 Cholelithiasis to serum total bilirubin

The identified IVs including the seven SNPs (as shown in **Table S2**) explained 3.21% of the variance of cholelithiasis. Moreover, the least F-statistic of these IVs was 33.02, suggesting that all IVs were effective enough for the MR analysis (F-statistic >10).

According to random-effect IVW estimates, the result supports causal effect of cholelithiasis to serum total bilirubin (beta = 0.12; 95% CI, 0.09 to 0.15; p < 0.001). At the same time, the other methods including the weighted median (beta = 0.10; 95% CI, 0.06 to 0.13; p < 0.001), MR-Egger (beta = 0.12; 95% CI, 0.07 to 0.18; p = 0.007), weighted mode (beta = 0.09; 95% CI, 0.03 to 0.14; p = 0.019), and MR-PRESSO methods further confirmed this result (p < 0.001) (as shown in **Table 1**). The scatter plot can be seen in **Figure 3C**.

The MR-Egger regression produced an intercept of −0.0007 with a p-value of 0.852 and the MR-PRESSO global test with a p-value of 0.077, indicating no significant horizontal pleiotropy in the selected SNPs. The forest plot of the leave-one-out result shows that no 95% CI of each selected SNP crosses the zero-line (**Figure 3D**). Although the Cochran Q’s test shows heterogeneity in our data (Q = 16.95, P < 0.05), our results remain robust because the multiplicative random-effect IVW was used.

## 4 Discussion

In the present study, bidirectional two-sample MR approaches were used for the first time to investigate whether serum total bilirubin and the risk of gallstone disease are causally associated. Our study showed that serum total bilirubin was causally associated with risk of gallstone disease. Meanwhile, we also found that the genetic predisposition to cholelithiasis is causally associated with the increased serum total bilirubin level.

Notably, rs6742078 is an SNP in the uridine diphosphate glucuronosyltransferase 1A1 (UGT1A1) gene (20). It was also the only SNP used in the study by Stender et al. Considering that the R^2^ of rs6742078 was 23.93% in our study, which indicated that it was a strong IV for plasma bilirubin, we included this SNP although it was filtered out of the SNP pool during the clumping process. It also passed the subsequent MR Steiger filtering and was not detected as an outlier in the MR-PRESSO procedure.

The potential impact of serum total bilirubin on cholelithiasis has been the subject of a number of studies. A case-control study conducted by Kitsiou-Tzeli et al. found that Gilbert syndrome was a predisposing factor for cholelithiasis risk in children (6). Another case-control study conducted by Tsezou et al. found that Gilbert syndrome was a predisposing factor for cholelithiasis risk in Greek adult (7). In addition, Gilbert syndrome is a common autosomal dominant hereditary condition with incomplete penetrance and characterized by intermittent unconjugated hyperbilirubinemia in the absence of hepatocellular disease or hemolysis (21). Hence, these two studies indicated that increased serum total bilirubin caused by Gilbert syndrome could be a risk factor of cholelithiasis. A prospective clinical and laboratory-based study carried by Shrestha et al. illustrated that Gilbert syndrome was associated with hyperbilirubinemia but not associated with cholelithiasis (9). However, this study was done in subjects with β-thalassemia and that 14 of the 102 subjects had cholelithiasis. Such a small sample size may indicate an insufficient statistical power.

Overall, observational studies are often criticized for containing flaws like confounding bias, random errors, and even causal inversion. In addition, the studies may not have enough statistical power because of the small sample sizes. Additional investigations into the results’ veracity and consistency are required.

Stender et al. observed that that extreme serum total bilirubin levels were associated with the risk of symptomatic gallstone disease, whereas bilirubin levels below the 10th decile were not associated with the risk of symptomatic gallstone disease. They also conducted an MR study that showed that extreme serum bilirubin levels were a causal risk factor for symptomatic gallstone disease (12). However, only one SNP (rs6742078) was used in their study, and no statistical pleiotropy test was conducted, which can be improved by our two-sample MR study. In addition, the MR study was processed with one sample. Therefore, our finding that there was causal association between serum total bilirubin and the risk of cholelithiasis was more compelling and was also an update and supplement of the study by Stender et al.

It should be noted that the definition of cholelithiasis in FinnGen is not quite the same as symptomatic gallstone disease in the study by Stender et al. Cholelithiasis in FinnGen GWAS is defined as ICD codes for cholelithiasis (ICD-8:574, ICD-9:574, and ICD-10:K80). In addition, symptomatic gallstone disease in the study by Stender et al. is defined as ICD codes for cholelithiasis or cholecystitis (ICD-8:574, ICD-8:575, ICD-10:K80, and ICD-10:K81). The condition that cholecystitis without cholelithiasis was included in their study. Hence, their definition was wider but not that strict.

On the other hand, the causal association of gallstone disease with the increased serum total bilirubin level can be explained as the obstructive jaundice, or cholestatic jaundice, to be specific, which is caused by the impacted stone in the cystic duct or bile duct (22). It is a common complications of gallstones and is associated with increased serum levels of bilirubin because of disruption of the functions of specific tranporters in the hepatocytes (23, 24). Our study proved that such association is causal.

Our MR study yielded a more reliable result for the following reasons. First, as one of the major strengths, the bidirectional two-sample MR method was used to evaluate the causal relationship between serum total bilirubin and the risk of gallstone disease in two GWAS studies whose sample sizes are much larger. Therefore, the statistical sensitivity of our method for identifying significant associations has been greatly improved in comparison to previous research. The second benefit of the bidirectional two-sample MR design is that it permits causal inference that is free from the problems of reverse causality and other confounding variables that can arise in observational studies. Last but not least, to determine the causal effects, we utilized not only the IVW method but also four complementary methods, namely, MR-Egger regression, weighted median, weighted mode, and MR-PRESSO. Consistent outcomes from each of these tests proved the validity and consistency of our findings.

Although our MR study had a number of advantages, there were also some limitations that needed to be taken into account. We were unable to investigate stratification effects based on sex, age, or other factors due to the GWAS summary-level statistics that we used. These factors could be investigated only when having the GWAS individual-level statistics. Moreover, cholelithiasis should be meticulously categorized so that the effect of serum total bilirubin on different types of gallstone formation can be investigated (25). Finally, we should be cautious when the results are interpreted in other populations, because the population of the included GWASs was only European.

In conclusion, our MR study revealed that the serum total bilirubin was causally associated with the risk of cholelithiasis, whereas the genetic predisposition to cholelithiasis is causally associated with the increased serum total bilirubin levels. Notably, given the limitations of our study, the causal association between serum total bilirubin and cholelithiasis needs further exploration.

## Data availability statement

The datasets presented in this study can be found in online repositories. The names of the repository/repositories and accession number(s) can be found in the article/**Supplementary Material**.

## Author contributions

Among the authors in the list, YS, JK, and SW designed the study and revised the manuscript. SY, WD, ZZ, XZ, YZ, JW, SB, and YD performed the literature search. YS drafted the manuscript. All authors contributed to the article and approved the submitted version.

## Supplementary material

Supplementary Figure 1 Forest plots of Wald ratio result, total bilirubin on cholelithiasis **(A)** and cholelithiasis on total bilirubin **(B).** Funnel plots of the relationship between genetically predicted total bilirubin on cholelithiasis **(C)** and cholelithiasis on total bilirubin **(D)**.
