# Supplementary figures and images for: Causal association between serum total bilirubin and cholelithiasis: a bidirectional two-sample Mendelian randomization study
Source: Front Endocrinol (Lausanne). 2023 Jul 4;14:1178486. doi: 10.3389/fendo.2023.1178486 (PMC10352914; doi:10.3389/fendo.2023.1178486)

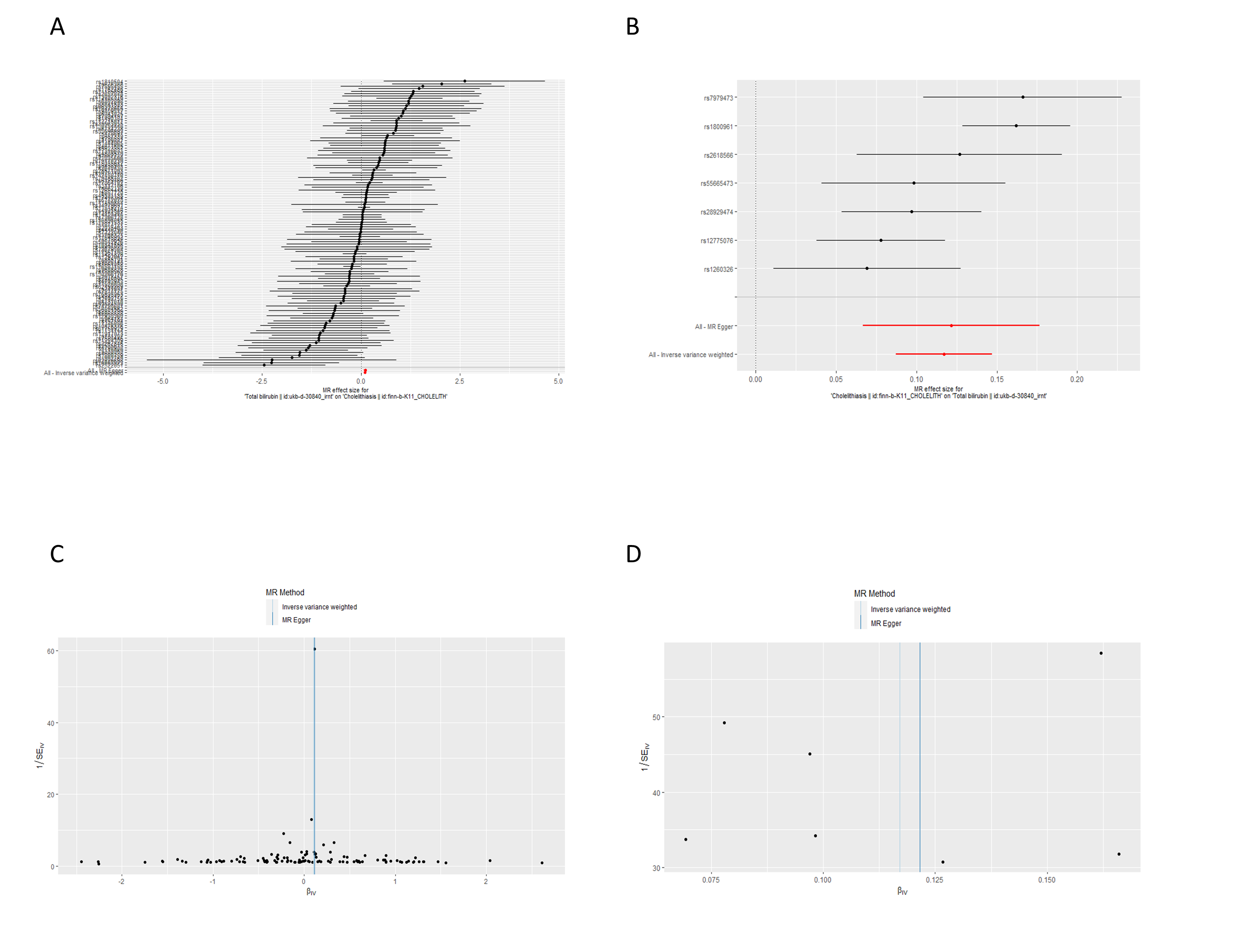

Supplement: Supplementary Figure 1 — Forest plots of Wald ratio result, total bilirubin on cholelithiasis (A) and cholelithiasis on total bilirubin (B). Funnel plots of the relationship between genetically predicted total bilirubin on cholelithiasis (C) and cholelithiasis on total bilirubin (D). [file Image_1.tif]
